# Intrapleural Fibrinolytic Therapy in the Management of Pediatric Pleural Empyema: A Narrative Review

**DOI:** 10.3390/ph19081207

**Published:** 2026-08-01

**Authors:** Susanna Esposito, Valentina Fainardi, Gaia Giorgia Arnesano, Nicola Principi

**Affiliations:** 1 Pediatric Clinic, Department of Medicine and Surgery, University Hospital of Parma, 43126 Parma, Italy; valentina.fainardi@unipr.it (V.F.); gaiagiorgia.arnesano@unipr.it (G.G.A.); 2 Department of Pathophysiology and Transplantation, Università degli Studi di Milano, 20122 Milan, Italy; nicola.principi@unimi.it

**Keywords:** pediatric empyema, parapneumonic effusion, fibrinolysis, urokinase, alteplase, tissue plasminogen activator, pleural infection, VATS

## Abstract

**Background:** Pediatric pleural empyema is a major complication of community-acquired pneumonia and remains associated with substantial morbidity despite advances in vaccination, antimicrobial therapy, and supportive care. Intrapleural fibrinolytic therapy has become an important minimally invasive treatment for complicated parapneumonic effusions and empyema, but uncertainty persists regarding the optimal fibrinolytic agent, treatment protocols, patient selection, and indications for surgical intervention. **Methods:** A narrative review of the literature was conducted to summarize current evidence on the use of intrapleural fibrinolytic therapy in pediatric pleural empyema. Experimental studies, randomized controlled trials, observational studies, systematic reviews, meta-analyses, and international clinical practice guidelines were critically reviewed. Particular attention was paid to the biological rationale for fibrinolysis, pharmacological characteristics of available agents, comparative effectiveness with video-assisted thoracoscopic surgery (VATS), practical treatment protocols, safety, and future research priorities. **Results:** Intrapleural fibrinolysis effectively improves pleural drainage by lysing fibrin septations during the fibrinopurulent stage of empyema and is associated with shorter hospitalization compared with chest-tube drainage alone. Urokinase remains the fibrinolytic agent supported by the highest-quality pediatric randomized evidence, whereas alteplase has demonstrated favorable outcomes in observational studies and randomized comparisons with VATS. Current evidence indicates comparable clinical outcomes between fibrinolysis and primary VATS in appropriately selected children, although fibrinolysis is generally associated with lower treatment costs and avoidance of surgery in most patients. Conventional-dose fibrinolytic therapy has an acceptable safety profile, with clinically significant bleeding reported only rarely. Current pediatric evidence does not support the routine addition of DNase to tissue plasminogen activator. **Conclusions:** Intrapleural fibrinolytic therapy represents a safe, effective, and minimally invasive first-line treatment for most children with complicated parapneumonic effusions and pleural empyema requiring drainage. Management should be individualized within a multidisciplinary framework, integrating timely diagnosis, image-guided pleural drainage, appropriate antimicrobial therapy, and selective surgical intervention. Future multicenter studies are needed to optimize fibrinolytic protocols, validate predictive biomarkers, and further standardize clinical management.

## 1. Introduction

Pleural empyema, defined as the accumulation of purulent fluid within the pleural cavity, represents the most advanced form of parapneumonic pleural infection and remains an important cause of morbidity among children hospitalized with community-acquired pneumonia [1]. Despite major advances in vaccination, antimicrobial therapy, diagnostic imaging, and supportive care, pediatric empyema continues to be associated with prolonged hospitalization, pleural drainage procedures, substantial healthcare resource utilization, and, in selected cases, surgical intervention [2].

During the past two decades, the epidemiology of pediatric empyema has changed substantially. The introduction of pneumococcal conjugate vaccines has markedly reduced disease caused by several vaccine serotypes and has lowered the incidence of pneumococcal parapneumonic effusion in a number of settings [3]. Nevertheless, the overall epidemiological effect has not been uniform. Persistent disease caused by residual or non-vaccine pneumococcal serotypes, together with an increasing contribution from Streptococcus pyogenes, Staphylococcus aureus, and other pathogens, has maintained a clinically relevant burden of complicated parapneumonic effusion and empyema in many countries [4]. Improvements in microbiological testing, pleural ultrasonography, and clinical awareness may also have contributed to more frequent and earlier recognition of the condition.

Management strategies have evolved considerably over the past three decades. Historically, treatment ranged from prolonged antimicrobial therapy and tube thoracostomy to open thoracotomy and decortication in advanced disease. The introduction of image-guided small-bore chest drainage, intrapleural fibrinolytic therapy, and video-assisted thoracoscopic surgery has transformed clinical practice by providing less invasive alternatives to open surgery [5].

Among these approaches, intrapleural fibrinolysis has gained widespread acceptance because it targets one of the principal pathological mechanisms underlying inadequate pleural drainage: fibrin deposition with the formation of septations and loculated collections. By promoting the enzymatic degradation of intrapleural fibrin, fibrinolytic agents improve communication between locules, facilitate evacuation of infected pleural fluid through an indwelling catheter, and allow many children to avoid operative intervention [6].

Nevertheless, several questions remain unresolved. Considerable interinstitutional variability persists regarding the preferred fibrinolytic agent, dosage, dilution volume, timing of administration, dwell time, chest tube size, treatment duration, and criteria for surgical referral. Furthermore, although combined intrapleural tissue plasminogen activator and deoxyribonuclease therapy improves selected outcomes in adults, a pediatric randomized trial did not demonstrate an additional benefit from DNase when compared with tissue plasminogen activator alone [2]. Pediatric evidence therefore continues to support chest tube drainage with a fibrinolytic agent alone as an appropriate first-line intervention in children requiring pleural drainage.

This review critically examines the evidence supporting intrapleural fibrinolytic therapy in pediatric empyema, with particular attention to its biological rationale, clinical efficacy, safety, practical administration, comparison with surgical management, and priorities for future research.

## 2. Methods

This narrative review was conducted to summarize and critically evaluate the available evidence on intrapleural fibrinolytic therapy for pediatric parapneumonic effusion and pleural empyema. The population of interest comprised infants, children, and adolescents aged from birth to 18 years. A structured literature search was performed in PubMed/MEDLINE, Embase, Scopus, and the Cochrane Library for publications available from database inception through May 2026. The literature search and evaluation were conducted between January 2000 and May 2026.

The search combined controlled vocabulary and free-text terms related to pediatric pleural infection and intrapleural treatment, including “infant,” “child,” “children,” “adolescent,” “pediatric,” “parapneumonic effusion,” “pleural empyema,” “pleural infection,” “fibrinolysis,” “urokinase,” “alteplase,” “tissue plasminogen activator,” “streptokinase,” “DNase,” “pleural lavage,” “thoracostomy,” and “video-assisted thoracoscopic surgery.” Reference lists of relevant reviews, clinical guidelines, randomized trials, and key observational studies were also examined to identify additional publications.

Eligible publications included studies enrolling participants younger than 18 years, including pediatric randomized controlled trials, prospective and retrospective observational studies, case series, systematic reviews, meta-analyses, pharmacological and experimental studies relevant to the biological rationale for fibrinolysis, and national or international clinical practice guidelines. Studies including both pediatric and adult participants were considered only when pediatric data were reported separately. Adult studies were included selectively when pediatric evidence was unavailable or when they provided important mechanistic, pharmacological, or comparative context, particularly regarding combined tissue plasminogen activator and DNase therapy, pleural lavage, and the pathophysiology of pleural organization.

Articles were excluded when they did not address pleural infection, did not provide information relevant to intrapleural therapy or its comparison with surgical management, or consisted solely of isolated case reports without additional clinical or mechanistic relevance. Titles and abstracts were reviewed for relevance, followed by full-text assessment of potentially eligible publications. Study selection prioritized pediatric-specific evidence, randomized and comparative studies, larger multicenter cohorts, systematic reviews, and current clinical guidelines.

Because this was a narrative rather than a systematic review, no formal risk-of-bias assessment or meta-analysis was undertaken. The included evidence was synthesized descriptively, with particular attention to the biological rationale for treatment, fibrinolytic agents and dosing regimens, clinical effectiveness, safety, comparison with video-assisted thoracoscopic surgery, practical administration, predictors of treatment response, guideline recommendations, and priorities for future research.

## 3. Epidemiology of Pediatric Pleural Empyema

Parapneumonic pleural effusions develop in approximately 20–40% of children hospitalized with bacterial pneumonia, although only a minority progress to complicated effusions or frank empyema requiring invasive intervention [7]. The reported incidence varies according to geographical region, vaccination coverage, referral patterns, and diagnostic criteria.

Population-based studies conducted in Europe, North America, and Australia have demonstrated a significant increase in pediatric empyema during the late 1990s and early 2000s, largely attributed to changing pneumococcal serotypes and improved detection [3,8]. Following introduction of the 7-valent and subsequently the 13-valent pneumococcal conjugate vaccines (PCV7 and PCV13), several countries reported a reduction in vaccine-serotype disease but persistence of empyema caused by emerging serotypes such as 3, 19A, and 1 before their inclusion in expanded vaccine formulations [9].

Current incidence estimates range from 2 to 12 cases per 100,000 children annually in developed countries, with the highest burden observed among preschool-aged children [10]. Boys appear to be affected slightly more frequently than girls, although sex differences are modest and probably not clinically significant.

The causative pathogens have also evolved over time. Streptococcus pneumoniae remains one of the most frequently identified pathogens and is the predominant organism in many, although not all, pediatric series [4,11]. Molecular diagnostic techniques have substantially increased pathogen detection compared with conventional culture, particularly in children receiving antibiotics before pleural fluid sampling. Other important pathogens include: Streptococcus pyogenes, methicillin-sensitive and methicillin-resistant Staphylococcus aureus, Haemophilus influenzae, anaerobic bacteria (rare in immunocompetent children), and mixed polymicrobial infections. Viral respiratory infections may predispose to bacterial superinfection but rarely cause empyema directly [12,13,14].

Several risk factors have been associated with progression from uncomplicated pneumonia to empyema, including delayed antibiotic treatment, highly virulent bacterial strains, host immune response, chronic neurological disease, immunodeficiency, and underlying pulmonary abnormalities. Nevertheless, most children who develop empyema are previously healthy, suggesting that pathogen virulence and inflammatory responses play more important roles than host comorbidities [11].

Mortality remains exceptionally low in high-income countries, generally below 1%, but morbidity remains substantial because hospitalization often exceeds one week and invasive procedures are frequently required [13].

## 4. Pathophysiology of Pleural Empyema

Understanding the biological evolution of pleural infection is fundamental to appreciating the rationale for intrapleural fibrinolytic therapy.

The pleural cavity normally contains only a thin layer of lubricating fluid maintained through a tightly regulated balance between pleural fluid production and lymphatic drainage [14]. During bacterial pneumonia, inflammatory mediators increase vascular permeability, allowing protein-rich fluid to accumulate within the pleural space.

The progression from uncomplicated parapneumonic effusion to organized empyema is traditionally divided into three overlapping stages originally described by the American Thoracic Society [15].

### 4.1. Exudative Stage

The initial exudative phase usually occurs during the first 24–72 h of infection. Increased capillary permeability leads to accumulation of sterile, free-flowing pleural fluid characterized by: low cellularity; relatively normal glucose; normal or mildly decreased pH; low viscosity. At this stage, antibiotic therapy alone may be sufficient because fibrin deposition has not yet occurred or is very limited [1].

### 4.2. Fibrinopurulent Stage

The second stage represents the critical window during which fibrinolytic therapy exerts its greatest benefit.

Ongoing pleural infection induces recruitment of neutrophils and macrophages and is associated with increased local concentrations of proinflammatory and profibrotic mediators, including interleukin-1, interleukin-6, tumor necrosis factor-α, and transforming growth factor-β. In parallel, increased plasminogen activator inhibitor-1 activity suppresses endogenous fibrinolysis and favors fibrin persistence [16,17]. As fibrinolytic activity decreases, fibrin accumulates on both visceral and parietal pleural surfaces, resulting in formation of multiple septations and loculated fluid collections [17]. These fibrinous adhesions have several important clinical consequences: compartmentalization of infected pleural fluid; impaired drainage through conventional chest tubes; persistence of bacterial infection; reduced antibiotic penetration; progressive restriction of lung expansion.

During the fibrinopurulent phase, thoracic ultrasonography may demonstrate echogenic debris, fibrin strands, septations, and multiloculated collections, thereby providing a practical assessment of pleural fluid complexity [18]. The fibrinopurulent phase therefore represents the ideal therapeutic target for intrapleural fibrinolytic agents, whose primary objective is enzymatic degradation of fibrinous septations, restoration of pleural fluid communication, and enhancement of chest tube drainage.

### 4.3. Organizing Stage

If infection persists, fibroblasts migrate into the pleural cavity under stimulation by transforming growth factor-β and platelet-derived growth factor. These cells produce collagen and extracellular matrix, progressively transforming fibrinous septa into dense fibrous tissue [19].

The visceral pleura becomes encased within a thick inelastic “pleural peel,” preventing normal lung re-expansion despite adequate evacuation of infected fluid. At this stage, pharmacological fibrinolysis becomes considerably less effective because collagen-rich fibrous tissue cannot be enzymatically lysed by plasmin activation alone.

Consequently, patients presenting with advanced organizing empyema may require surgical decortication to restore pulmonary function [20].

**Figure 1:**
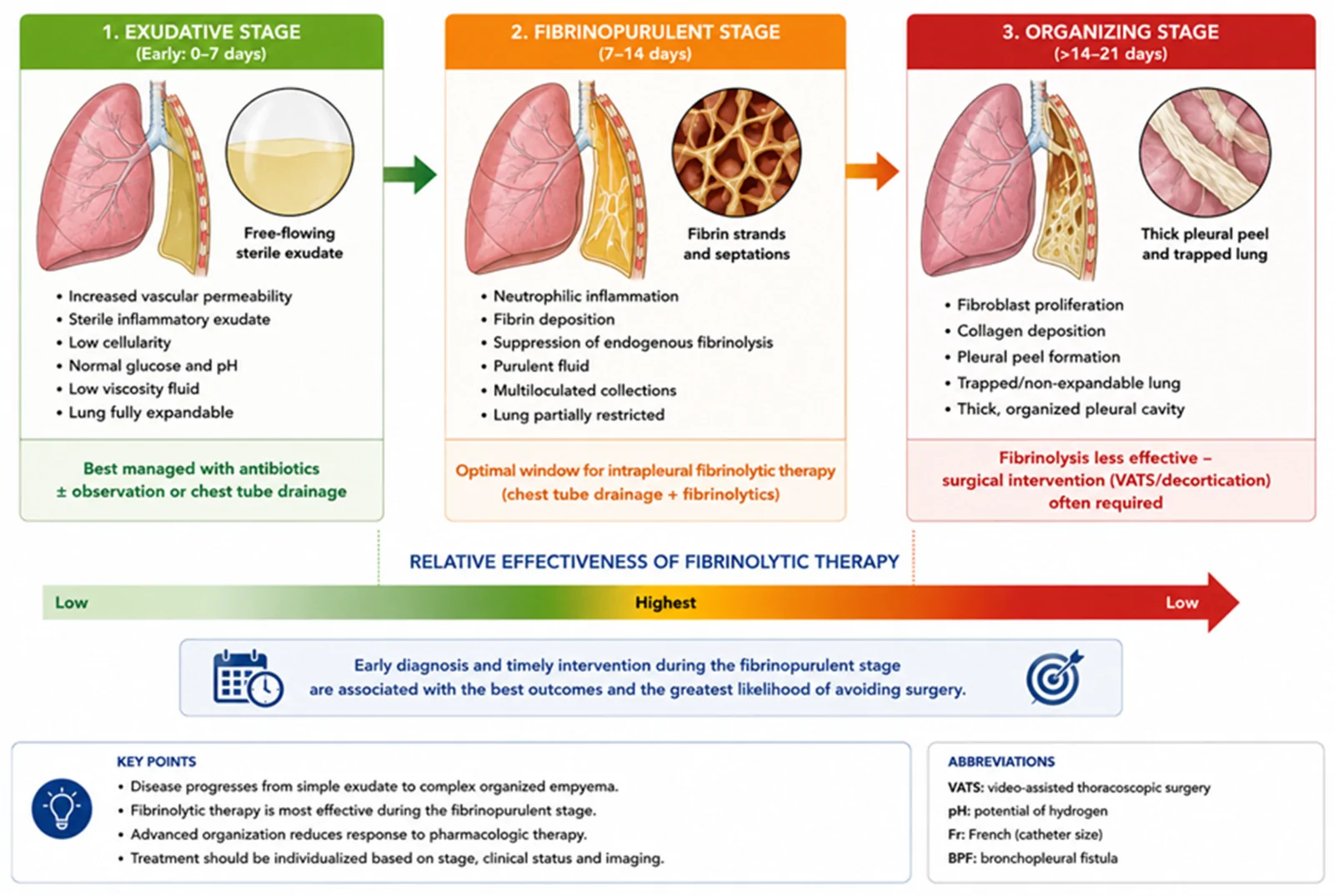
A schematic illustration of the pathophysiological progression from exudative to fibrinopurulent to organizing empyema, highlighting the point at which fibrinolytic therapy is most effective.

The sequential evolution of parapneumonic pleural infection and the changing effectiveness of intrapleural fibrinolytic therapy throughout the different pathological stages are illustrated in Figure 1.

## 5. Biological Rationale for Intrapleural Fibrinolytic Therapy

The development of fibrinous septations during the fibrinopurulent stage is an important cause of failure of simple pleural drainage. Pharmacological degradation of these septations has therefore become a rational therapeutic strategy for children with complicated parapneumonic effusions and empyema [6].

Under physiological conditions, fibrin turnover is regulated by a balance between coagulation and fibrinolysis. Tissue plasminogen activator and urokinase-type plasminogen activator convert plasminogen into plasmin, the principal protease responsible for fibrin degradation. Plasmin cleaves fibrin into soluble degradation products and contributes to the resolution of fibrin deposited after pleural injury [21].

During pleural infection, this balance shifts toward a procoagulant and antifibrinolytic state. Human and experimental studies have demonstrated increased tissue-factor activity, local thrombin generation, fibrin deposition, and suppression of endogenous fibrinolysis through increased activity of plasminogen activator inhibitor-1 [22]. High pleural fluid PAI-1 concentrations have been associated with the presence and severity of sonographic septations, as well as with longer hospitalization and increased mortality in adult pleural infection [23].

As fibrin accumulates, septa may divide the pleural cavity into partially or completely isolated compartments, limiting effective drainage through a single catheter. Infected pleural fluid may also become highly viscous because of inflammatory cells, cellular debris, protein-rich material, bacterial products, and extracellular DNA released during neutrophil breakdown [24].

Unlike surgery, which mechanically removes pus and disrupts adhesions, fibrinolytic therapy acts by promoting local plasmin generation and degrading accessible fibrinous septations. This process may restore communication between locules and improve evacuation of infected pleural fluid through an appropriately positioned chest drain [25].

Fibrinolytic agents have no direct antibacterial activity. Their clinical efficacy therefore depends on appropriate antimicrobial therapy, satisfactory catheter positioning, maintenance of drain patency, and adequate pleural evacuation. Intrapleural fibrinolysis should consequently be regarded as an adjunct to antibiotics and drainage rather than as an alternative to either intervention [1].

The biological rationale for fibrinolysis is strongest during the fibrinopurulent phase, before fibrinous adhesions undergo extensive cellular invasion and collagen deposition. As pleural organization progresses, fibroblasts deposit extracellular matrix and convert fibrin-rich septa into mature fibrous tissue. Because collagenous adhesions are less susceptible to plasmin-mediated degradation, pharmacological fibrinolysis becomes less likely to succeed in advanced organizing empyema [17].

Although pleural infection is conventionally divided into exudative, fibrinopurulent, and organizing stages, these phases represent a biological continuum rather than sharply separated entities. In particular, the late fibrinopurulent and early organizing phases may overlap substantially and are often difficult to distinguish on the basis of symptom duration, imaging findings, or pleural fluid characteristics alone. Therefore, staging in clinical practice is necessarily approximate, and treatment decisions should integrate the child’s overall clinical condition, the complexity and drainability of the pleural collection, and the response to initial therapy rather than rely on a rigid stage classification.

## 6. Available Fibrinolytic Agents

Several fibrinolytic agents have been evaluated for intrapleural administration in children. Although streptokinase, urokinase, and alteplase all increase plasmin-mediated fibrin degradation, they differ in their mechanisms of plasminogen activation, fibrin specificity, immunogenicity, availability, dosing experience, and strength of pediatric evidence.

### 6.1. Streptokinase

Streptokinase was one of the earliest fibrinolytic agents used in pleural infection. It is a bacterial protein produced by several strains of β-hemolytic streptococci. Streptokinase has no intrinsic protease activity; instead, it forms an activating complex with plasminogen, which subsequently promotes the conversion of additional plasminogen molecules into plasmin [26].

The historical advantages of streptokinase included relatively low cost and broad availability. Its limitations, however, have substantially reduced its role in modern practice. Streptokinase is not fibrin-specific and can promote plasmin generation in both clot-bound and circulating plasminogen. In addition, its bacterial origin makes it immunogenic. Previous streptococcal infection or earlier exposure to streptokinase may result in neutralizing antibodies, reduced fibrinolytic activity, fever, or hypersensitivity reactions [27].

The large adult Multicenter Intrapleural Sepsis Trial, generally referred to as MIST1, found that intrapleural streptokinase did not reduce mortality, the need for surgery, radiographic abnormalities, or hospital length of stay compared with placebo [28]. Pediatric evidence is also unconvincing. A randomized pediatric trial found no significant short-term clinical or ultrasonographic benefit from intrapleural streptokinase, although a possible reduction in subsequent pleural thickening was reported in a subgroup with multiloculated disease [29]. Streptokinase should therefore not be regarded as a preferred pediatric fibrinolytic when urokinase or alteplase is available.

### 6.2. Urokinase

Urokinase, the two-chain form commonly referred to in the literature, has the strongest direct randomized-trial evidence among fibrinolytic agents used for pediatric empyema. Urokinase-type plasminogen activator is a human serine protease that directly cleaves plasminogen to generate plasmin. Unlike streptokinase, it does not require formation of an antigenic bacterial protein–plasminogen complex and is therefore associated with substantially less immunogenicity [25].

Clinical advantages of urokinase include extensive pediatric experience, low antigenicity, favorable intrapleural tolerability, and a standardized regimen supported by a pediatric randomized trial [30]. Although urokinase is not as fibrin-selective as tissue plasminogen activator, clinically important systemic coagulation disturbances have been uncommon after intrapleural administration in children. This observation should not, however, be interpreted as proof that systemic absorption is absent [30].

In the landmark multicenter randomized trial conducted by Thomson and colleagues, children receiving intrapleural urokinase had a shorter hospital stay than those receiving saline. The greatest benefit was observed when urokinase was administered through a small-bore percutaneous drain. No important excess of adverse events was identified [6]. These findings formed the principal evidence supporting the British Thoracic Society recommendation that urokinase be used in children with complicated, loculated parapneumonic effusions or empyema treated by chest-tube drainage [1]. The British Thoracic Society guideline recommended intrapleural fibrinolytic therapy for children with complicated parapneumonic effusions, defined by thick or loculated pleural fluid, and for those with frank empyema [1]. Thus, urokinase was not restricted to rescue treatment after failure of chest-tube drainage or to cases in which limited drainage coexisted with persistent pleural sepsis. Rather, it was recommended as an adjunct to chest-tube drainage from the outset when the pleural collection was loculated or purulent and therefore unlikely to drain adequately through a catheter alone. Inadequate drainage and ongoing sepsis despite correctly positioned drainage and fibrinolytic therapy were indications for reassessment and possible surgical intervention.

Subsequent observational studies have generally supported small-bore catheter drainage with urokinase as an effective first-line strategy. However, reported success rates vary according to disease stage, definitions of treatment failure, catheter technique, and thresholds for surgical referral. It is therefore preferable to state that most children in published series avoided additional operative intervention rather than assigning a universal success rate of 80–95% [31].

### 6.3. Alteplase

Alteplase is a recombinant form of human tissue-type plasminogen activator and has become the most frequently used intrapleural fibrinolytic in many North American pediatric centers, partly because urokinase has not always been readily available. Alteplase preferentially activates plasminogen in the presence of fibrin and is therefore relatively fibrin-selective, although this selectivity is not absolute and does not eliminate the possibility of local or systemic bleeding [32].

There is no universally accepted pediatric alteplase regimen. Published protocols include fixed doses of 2–4 mg, weight-based doses near 0.1 mg/kg, and substantially higher regimens of up to 0.4 mg/kg or 10 mg per dose. Alteplase is generally diluted in normal saline, administered through the pleural catheter, and left within the pleural space for approximately 30–80 min before drainage is resumed. Once-daily treatment for three doses is supported by prospective pediatric trial experience, but other schedules have also been reported [33].

Observational pediatric studies indicate that alteplase increases pleural drainage and allows many children to recover without surgery. In one cohort, children receiving early or delayed alteplase had greater pleural fluid drainage than children treated with tube drainage alone [34]. The reported effect of alteplase primarily referred to an increase in pleural fluid output through the thoracostomy tube rather than to a formally quantified improvement on chest imaging. This finding should be interpreted cautiously because increased drainage after tPA administration may reflect not only disruption of fibrinous septations and improved communication among locules, but also tPA-associated stimulation of pleural fluid formation, as suggested by preclinical studies. Consequently, greater chest-tube output alone should not be considered proof of superior pleural clearance, and the clinical response should be assessed together with respiratory status, inflammatory markers, and follow-up imaging when indicated [34]. Nevertheless, safety and efficacy should not be overstated. A retrospective study using a median alteplase dose of 7 mg reported avoidance of surgery in 84% of patients, but pain and oxygen desaturation were common, and 12% of children received a blood transfusion during treatment. The retrospective design did not establish that all transfusions were caused by alteplase, but the findings emphasize the need for clinical and hematologic monitoring when higher doses are used [33].

The prospective randomized trial by St Peter and colleagues compared a fixed intrapleural alteplase regimen with primary video-assisted thoracoscopic surgery. Hospital stay, duration of fever, oxygen requirement, and time to recovery were similar between groups. Fibrinolysis was associated with lower hospital charges, although some children required rescue surgery [35]. The study supports alteplase-based fibrinolysis as a reasonable first-line alternative to primary VATS rather than demonstrating that alteplase is intrinsically superior to surgery.

### 6.4. Tenecteplase and Other Investigational Agents

Tenecteplase is a genetically modified tissue plasminogen activator with a longer circulating half-life, greater fibrin selectivity, and increased resistance to plasminogen activator inhibitor-1 compared with alteplase [36]. These properties are advantageous for systemic thrombolysis but do not necessarily predict superior efficacy or safety after intrapleural administration.

Published intrapleural tenecteplase evidence consists principally of small adult studies and case-based experience. One uncontrolled adult study reported improved drainage of loculated parapneumonic effusions and empyema after tenecteplase administration, but the study was not pediatric and did not establish superiority over urokinase, alteplase, or surgery [37]. Robust pediatric dosing, pharmacokinetic, efficacy, and safety data are lacking. Tenecteplase should therefore be described as an investigational intrapleural agent and should not be recommended routinely for pediatric empyema.

Evidence for other recombinant plasminogen activators is even more limited. None has demonstrated a clear pediatric advantage over urokinase or alteplase.

### 6.5. DNase as an Adjunct Rather than a Fibrinolytic Agent

Recombinant human deoxyribonuclease, or dornase alfa, is not a fibrinolytic agent. Its proposed role in pleural infection is based on the presence of large quantities of extracellular DNA in purulent fluid, much of it released by degenerating neutrophils. Extracellular DNA contributes substantially to the high viscosity of pus. In vitro studies have demonstrated that DNase can markedly reduce the viscosity of empyema fluid, whereas fibrinolytic therapy primarily targets fibrinous septations [38].

In the adult MIST2 trial, combined intrapleural alteplase and DNase improved radiographic pleural clearance, reduced surgical referral, and shortened hospital stay compared with placebo. Neither alteplase alone nor DNase alone produced the same overall benefit [24]. These findings established the combination as an important treatment option in adult pleural infection but could not automatically be extrapolated to children.

An initial pediatric pilot study suggested that combined tPA and DNase was feasible and did not produce major treatment-related complications in a small group of children [39]. More importantly, a subsequent multicenter randomized, placebo-controlled trial enrolled 97 children and compared three daily doses of 4 mg tPA followed by 5 mg DNase with tPA followed by saline. The addition of DNase did not reduce hospital length of stay, time to chest-tube removal, fever duration, additional drainage procedures, readmission, or healthcare costs [2].

Current pediatric evidence therefore does not support the routine addition of DNase to intrapleural tPA [2]. Chest-tube drainage with a fibrinolytic agent alone remains an appropriate first-line pharmacological strategy. DNase may still be considered in unusual or refractory cases after multidisciplinary assessment, but such use cannot presently be regarded as evidence-based standard pediatric therapy.

The reasons why DNase provides additional benefit in adults but not in children remain uncertain. One possible explanation is that pediatric pleural infection is often recognized and treated earlier, when collections may be less organized and fibrin degradation with tPA alone is sufficient to restore drainage [39]. In contrast, adults enrolled in trials such as MIST2 frequently had a longer duration of illness and more mature, viscous, and organized pleural collections, in which degradation of extracellular DNA may provide greater additional benefit [24]. Age-related differences in pleural inflammatory responses, comorbidity burden, microbiology, and the composition of empyema fluid may also contribute, but these hypotheses have not been directly tested.

The principal pharmacological characteristics, advantages, limitations, and current evidence supporting the available intrapleural agents are summarized in Table 1.

## 7. Pharmacokinetics and Safety Considerations

An important consideration when using intrapleural fibrinolytic therapy in children is the possibility of local bleeding or systemic fibrinolytic effects [40]. Direct pediatric pharmacokinetic data are limited, and the extent of systemic absorption after intrapleural administration has not been adequately quantified. Intrapleural treatment is nevertheless intended to achieve a predominantly local effect, using doses that are generally lower than those employed for systemic thrombolysis. Local binding, inactivation within pleural fluid, and clearance from the pleural space may further limit systemic exposure, although systemic absorption cannot be assumed to be absent [40].

Most pediatric reports using urokinase or conventional-dose alteplase have not identified clinically important systemic coagulation abnormalities. Available studies and clinical series have generally reported no consistent treatment-related changes in prothrombin time, activated partial thromboplastin time, fibrinogen concentration, or platelet count [34]. However, laboratory monitoring has not been standardized across studies, and many investigations were not designed specifically to detect systemic fibrinolysis [34]. Routine measurement of coagulation parameters before and after every dose is therefore not supported by strong evidence in otherwise healthy children, but baseline testing and additional monitoring are appropriate when bleeding risk factors are present.

Blood-stained pleural drainage is relatively common after fibrinolytic administration and is usually mild and self-limiting. Minor hemorrhagic discoloration of the drained fluid, in the absence of hemodynamic instability or a substantial fall in hemoglobin, does not necessarily indicate clinically significant bleeding. Pain during instillation and transient oxygen desaturation are also reported adverse effects, particularly with larger alteplase doses.

Major hemorrhage appears uncommon, but its exact frequency in children cannot be reliably estimated from the available literature. Most pediatric trials and case series have reported few serious bleeding events, although clinically significant intrapleural hemorrhage requiring blood transfusion has been described. In a retrospective series of 73 children treated with relatively large alteplase doses, 12% received a blood transfusion during admission, although the investigators could not establish that all transfusions were caused by fibrinolytic therapy. The same study reported mostly minor pleural bleeding, pain, and transient oxygen desaturation. These findings indicate that bleeding risk may depend on the dose employed, underlying pulmonary necrosis, concomitant coagulopathy, and other clinical factors [33].

Intrapleural fibrinolysis should generally be avoided in children with active major bleeding, severe uncorrected coagulopathy, clinically important thrombocytopenia, or a documented serious hypersensitivity to the selected agent. Recent major surgery accompanied by ongoing or inadequately controlled bleeding also constitutes a strong contraindication. Recent invasive procedures, congenital bleeding disorders, therapeutic anticoagulation, severe hepatic dysfunction, and extensive necrotizing pulmonary disease should be regarded as relative contraindications requiring individualized assessment rather than automatic exclusion from treatment [1]. Specific pediatric evidence regarding concomitant prophylactic-dose heparin and intrapleural fibrinolytic therapy is lacking. Prophylactic anticoagulation should not necessarily be regarded as an absolute contraindication; however, because an additive bleeding risk is biologically plausible, the indication for heparin should be reviewed and management individualized according to thrombotic and hemorrhagic risk [1]. When clinically feasible, temporary withholding of prophylactic heparin around fibrinolytic administration may be considered, particularly in children with additional bleeding risk factors, with close monitoring of pleural drainage, hemoglobin concentration, and clinical signs of hemorrhage. Therapeutic anticoagulation warrants greater caution and multidisciplinary assessment.

Before treatment, clinicians should review the child’s bleeding history, current medications, platelet count, and relevant coagulation results when risk factors are present. During therapy, monitoring should include the appearance and volume of pleural drainage, catheter-site bleeding, pain, respiratory status, hemodynamic stability, and hemoglobin concentration when clinically indicated. A sudden increase in heavily blood-stained drainage, hemodynamic deterioration, an unexplained fall in hemoglobin, or evidence of bleeding at another site should prompt suspension of fibrinolytic administration and urgent reassessment.

Overall, intrapleural urokinase and conventional-dose alteplase have demonstrated favorable safety profiles in appropriately selected children. Nevertheless, the absence of uniform dosing protocols and standardized adverse-event reporting means that fibrinolytic therapy should not be described as risk-free. Careful patient selection, appropriate dosing, correct catheter placement, and clinical monitoring remain essential.

## 8. Evolution of Clinical Evidence

Although the biological rationale for intrapleural fibrinolysis is compelling, therapeutic recommendations must ultimately be based on clinically meaningful outcomes. Early experience consisted principally of case reports, small uncontrolled series, and retrospective comparisons describing the use of streptokinase or urokinase in patients with loculated pleural infection. These reports suggested that fibrinolytic administration could increase pleural fluid drainage, improve radiographic appearances, and reduce the need for open thoracotomy, but their methodological limitations prevented firm conclusions regarding efficacy [41].

The uncontrolled nature of the early literature was particularly important because many children with empyema recover with appropriate antimicrobial therapy and effective pleural drainage, irrespective of the adjunctive intervention selected. Differences in disease severity, timing of drainage, catheter size and position, fibrinolytic dose, and institutional thresholds for surgical referral further complicated comparisons between published series [41].

Recognition of these limitations led to pediatric randomized controlled trials evaluating intrapleural fibrinolysis against placebo and, subsequently, against primary video-assisted thoracoscopic surgery. These studies transformed fibrinolysis from an empirically used adjunct into an evidence-supported treatment option for children requiring pleural drainage [41].

Pleural lavage with normal saline has also been evaluated in children, although the evidence is much more limited than in adults. In a small prospective randomized crossover study of children with complicated parapneumonic effusion, saline irrigation was used as an active comparator to intrapleural alteplase; alteplase produced greater thoracostomy-tube drainage and a larger reduction in pleural fluid volume on computed tomography [34]. Thus, saline lavage appeared feasible and was not associated with major complications, but the study did not establish it as an effective stand-alone alternative to fibrinolytic therapy. In contrast to adult practice, where a randomized trial suggested that saline irrigation may improve pleural clearance and reduce surgical referral, dedicated pediatric trials evaluating clinically relevant outcomes are lacking. Pleural lavage therefore cannot currently be recommended as standard therapy for pediatric empyema and should be considered investigational or reserved for selected circumstances when established approaches are unsuitable.

### 8.1. Clinical Evidence Supporting Intrapleural Fibrinolytic Therapy

The role of intrapleural fibrinolysis in pediatric empyema has evolved progressively and is now supported by randomized trials, observational cohorts, systematic reviews, and meta-analyses. The available evidence indicates that fibrinolytic therapy can improve pleural drainage and may shorten hospitalization compared with chest-tube drainage alone. Comparative studies also suggest that drainage with fibrinolysis and primary VATS achieve broadly similar clinical outcomes in many children, although the certainty of evidence remains limited by relatively small trial populations and substantial heterogeneity among treatment protocols [42].

The principal outcomes assessed in pediatric studies include length of hospitalization, duration of fever and oxygen therapy, duration of chest-tube drainage, need for an additional pleural procedure, conversion to surgery, radiographic resolution, treatment-related complications, and hospital costs. Long-term respiratory outcomes have also been evaluated in some comparative studies. Because definitions of treatment success and failure vary among institutions, numerical success rates should be interpreted cautiously.

Overall, the evidence supports chest-tube drainage combined with a fibrinolytic agent as a reasonable first-line strategy in children with complicated or loculated parapneumonic effusions. This conclusion does not imply that fibrinolysis is appropriate for every child or that it is superior to surgery in all circumstances. Patient selection, disease stage, catheter position, local expertise, and access to pediatric thoracic surgery remain important determinants of outcome.

### 8.2. Early Clinical Studies

Before pediatric randomized trials became available, studies in adults provided proof of concept that enzymatic disruption of fibrinous septations could improve drainage of complicated pleural collections. In a randomized, double-blind adult trial, Bouros and colleagues compared intrapleural urokinase with saline in patients with complicated parapneumonic effusions or empyema. Urokinase was associated with greater fluid drainage and radiographic improvement, without evidence of clinically important systemic fibrinolysis [43]. Although this study did not include a pediatric population, it contributed to the rationale for subsequent trials in children.

Early pediatric experience was derived from institutional series in which chest-tube drainage with a fibrinolytic agent was incorporated into treatment algorithms intended to avoid thoracotomy. Meier and colleagues reported a retrospective pediatric management experience that included fibrinolytic therapy as part of a less invasive approach to empyema [44]. Other pediatric series subsequently described favorable outcomes with urokinase, including improved drainage and avoidance of operative intervention in many children [30].

These studies demonstrated feasibility but could not reliably establish comparative effectiveness. Their interpretation was limited by small sample sizes, retrospective designs, nonstandardized treatment regimens, historical control groups, and differences in the severity and stage of empyema. Claims that early observational studies consistently produced rapid normalization of inflammatory markers or success rates above a specific threshold should therefore be avoided.

### 8.3. The Thomson Randomized Controlled Trial

The multicenter randomized trial conducted by Thomson and colleagues provided the most important placebo-controlled pediatric evidence for intrapleural urokinase [6]. Sixty children with parapneumonic empyema were recruited from ten centers and randomly assigned, under double-blind conditions, to receive either intrapleural urokinase or saline. Urokinase was administered every 12 h for three days. Children aged one year or older received 40,000 IU diluted in 40 mL of saline, whereas infants received 10,000 IU diluted in 10 mL.

The prespecified primary outcome was hospital length of stay after entry into the trial. Children assigned to urokinase had a significantly shorter hospital stay than those receiving saline, with geometric mean stays of 7.4 and 9.5 days, respectively. The shortest hospitalization was observed among children treated with the combination of urokinase and a small percutaneous chest drain. No important excess of treatment-related adverse events was identified [6].

The trial should not, however, be interpreted as demonstrating statistically significant improvement in every secondary outcome. Its most robust finding was the reduction in hospital length of stay. The study nevertheless provided direct evidence that urokinase conferred benefit beyond saline instillation and conventional drainage alone.

Because it was pediatric-specific, multicenter, randomized, double-blind, and placebo-controlled, the Thomson trial strongly influenced subsequent British Thoracic Society guidance. The BTS consequently recommended intrapleural fibrinolytic therapy for children with complicated parapneumonic effusions or empyema and identified urokinase as the agent supported by the strongest pediatric trial evidence [1].

### 8.4. Alteplase Experience

Alteplase became increasingly used in North American pediatric centers, particularly during periods when urokinase was unavailable. The initial evidence consisted mainly of case reports and retrospective observational cohorts rather than placebo-controlled trials.

Weinstein and colleagues compared children receiving early or delayed intrapleural alteplase with a historical group managed using chest-tube drainage alone. Twelve children received alteplase within 24 h of diagnosis, 18 received it later, and 23 underwent drainage without alteplase. Alteplase-treated children demonstrated increased pleural fluid drainage, and no local or systemic bleeding was recorded in that cohort. However, the nonrandomized design, historical controls, and differences in treatment timing limited causal interpretation [34].

Later retrospective studies confirmed that alteplase can increase chest-tube output and allow many children to avoid surgery, but they also demonstrated considerable variation in dose, dwell time, number of administrations, and adverse-event reporting. In a series of 73 patients treated with a relatively large-dose protocol, chest-tube output increased significantly after alteplase and 84% did not undergo subsequent surgery [33]. The large-dose alteplase protocol used an initial intrapleural dose of 0.4 mg/kg, with a maximum of 10 mg per administration. Alteplase was diluted in normal saline, instilled through the thoracostomy tube, and retained in the pleural space for approximately 80 min before drainage was resumed. Additional doses were administered according to the clinical and radiographic response rather than according to a uniform fixed schedule. In the retrospective cohort, the median administered dose was approximately 7 mg. Although chest-tube output increased and most children avoided surgery, pain, transient oxygen desaturation, pleural bleeding, and blood transfusion were reported, and this regimen has not been shown to be more effective than conventional lower-dose protocols [33].

The existing literature therefore supports alteplase as an effective intrapleural fibrinolytic option, but it does not establish a universal success rate or an optimal dosing regimen. No adequately powered randomized pediatric trial has directly compared alteplase with urokinase. Assertions that the two agents have equivalent efficacy and safety are consequently based on indirect comparisons across heterogeneous studies rather than head-to-head evidence.

Serious hemorrhage appears uncommon with conventional-dose regimens, but clinically significant intrapleural bleeding has been reported. Safety conclusions should therefore take account of the alteplase dose, pulmonary necrosis, coagulation abnormalities, and concomitant bleeding risks rather than treating all published regimens as interchangeable.

The pivotal clinical trials that have shaped current pediatric practice are summarized in Table 2.

## 9. Fibrinolysis Versus Video-Assisted Thoracoscopic Surgery

One of the most clinically relevant questions in pediatric empyema is whether chest-tube drainage with intrapleural fibrinolysis should be preferred to early video-assisted thoracoscopic surgery. VATS permits direct visualization of the pleural cavity, evacuation of purulent material, mechanical disruption of loculations, debridement of fibrinous material, and, when necessary, limited decortication. These theoretical advantages led many surgeons to advocate early thoracoscopy as definitive treatment, recognizing the risks of protracted chest pain, scarring, and surgical or anesthesia complications.

Randomized evidence, however, has not consistently demonstrated clinically important superiority of primary VATS over chest-tube drainage with fibrinolysis. The principal trials have generally found similar durations of hospitalization, fever, oxygen requirement, and chest-tube drainage, although VATS may reduce the need for an additional pleural intervention in some populations [35,45,46].

On the basis of the available pediatric evidence, we favor chest-tube drainage with intrapleural fibrinolytic therapy as the initial invasive strategy for most children with complicated parapneumonic effusion or empyema who require drainage, provided that the child is clinically stable and effective catheter placement can be achieved. Surgery should generally be reserved for patients with persistent sepsis or respiratory compromise, inadequate drainage despite a correctly positioned catheter and fibrinolysis, advanced pleural organization, or complications requiring operative management. This stepwise approach is consistent with contemporary adult practice, although treatment selection should remain individualized according to disease severity, local expertise, and the availability of pediatric surgical services.

### 9.1. The St Peter Randomized Trial

St Peter and colleagues conducted a prospective randomized trial comparing primary thoracoscopic decortication with tube thoracostomy and intrapleural alteplase in children with empyema [35]. Thirty-six children were randomized to one of the two treatment strategies. In the fibrinolysis group, alteplase was administered at a dose of 4 mg at the time of chest-tube placement and repeated 24 and 48 h later. The investigators assessed hospital length of stay after intervention, duration of oxygen therapy, time to defervescence, analgesic use, treatment failure, complications, and hospital charges. No statistically significant differences were observed between the groups in post-treatment hospitalization, duration of fever, oxygen requirement, or analgesic use. Three of the eighteen children assigned to fibrinolysis subsequently underwent thoracoscopic surgery because of an inadequate clinical response. Hospital charges were significantly higher in the VATS group, principally because of operating-room and procedural expenses. One child assigned to VATS experienced an acute clinical deterioration requiring temporary ventilatory and circulatory support, whereas no major complications were reported in the fibrinolysis group [35]. The trial therefore demonstrated that initial tube drainage with alteplase achieved clinical outcomes similar to those of primary VATS in most children while reducing initial treatment costs. However, its small sample size limited its ability to detect uncommon adverse events or modest differences in treatment success.

### 9.2. The Sonnappa Randomized Trial

Sonnappa and colleagues prospectively randomized children with pleural empyema to primary VATS or percutaneous chest drainage with intrapleural urokinase [45]. The primary endpoint was the number of hospital days following the intervention. Secondary endpoints included chest-drain duration, total hospital stay, treatment failure, radiographic outcome at six months, and total treatment costs. No significant differences were identified in the principal clinical outcomes. Hospital stay after intervention, total hospitalization, duration of chest drainage, treatment-failure rate, and radiographic resolution were similar between the two groups. Follow-up also demonstrated satisfactory recovery in both treatment arms, without evidence that primary VATS produced a superior medium-term respiratory outcome. The urokinase strategy was less costly than primary surgery. The investigators therefore concluded that chest drainage with urokinase and primary VATS were similarly effective, while urokinase represented the more economical initial approach [45]. The results support individualized treatment based on clinical circumstances, local expertise, availability of interventional radiology and pediatric surgery, and institutional costs rather than an assumption that one intervention is universally superior.

### 9.3. Additional Randomized Evidence

A subsequent multicenter randomized trial by Marhuenda and colleagues compared drainage with urokinase against VATS in 103 children with septated parapneumonic empyema [46]. This was larger than the earlier St Peter and Sonnappa trials. Length of stay after treatment, total hospitalization, duration of fever, chest-drain duration, and treatment-failure rates were not significantly different between the groups. These findings further supported both treatments as acceptable initial strategies for septated pediatric empyema.

An earlier small randomized study by Kurt and colleagues compared primary VATS with conventional thoracostomy drainage. Children undergoing VATS had shorter hospitalization and fewer subsequent imaging and interventional procedures [47]. However, the comparator was not a uniform modern fibrinolysis protocol, only 18 children were enrolled, and subsequent therapies were permitted in the drainage group. The study therefore should not be interpreted as definitive evidence that VATS is superior to chest drainage combined with standardized fibrinolysis.

### 9.4. Observational and Comparative Studies

Observational studies have generally confirmed that most children treated initially with chest drainage and fibrinolysis recover without open surgery. Gates and colleagues reviewed 54 children managed with drainage, fibrinolysis, surgery, or combinations of these treatments. Approximately 80% were successfully managed without operative intervention, whereas 11 children ultimately required surgery [48]. The study also illustrated the difficulty of comparing treatment strategies retrospectively because children selected for surgery may have more advanced disease.

Livingston and colleagues subsequently examined outcomes at a center that changed its preferred initial strategy from VATS to chest-tube drainage with fibrinolysis. The transition did not result in an evident deterioration in major clinical outcomes, supporting fibrinolysis as a practical first-line approach within an appropriately organized treatment pathway [49].

These studies do not justify a universal claim that fibrinolysis succeeds in more than 85% of cases. Reported conversion rates depend on the fibrinolytic agent and dose, disease severity, timing of referral, catheter placement, local surgical practice, and the definition of treatment failure. Mortality is very low in contemporary pediatric series, but the available studies are generally underpowered to compare mortality between interventions.

Evidence regarding predictors of fibrinolytic failure is also inconsistent. Inadequate catheter position, persistent undrained locules, prolonged symptoms, necrotizing pneumonia, bronchopleural fistula, and advanced pleural organization may contribute to failure, but no imaging feature or laboratory marker reliably identifies all children who require surgery.

The assertion that unsuccessful fibrinolysis never complicates subsequent surgery should be phrased cautiously. Published trials have not demonstrated a clear adverse effect of an initial fibrinolytic strategy on eventual recovery, and rescue VATS is generally feasible. Nevertheless, the evidence is insufficient to conclude that delay is harmless in every child. Persistent sepsis, respiratory deterioration, or failure of adequate drainage requires prompt reassessment and timely surgical consultation.

### 9.5. Systematic Reviews and Meta-Analyses

A systematic review of randomized pediatric trials by Mahant and colleagues found no convincing evidence that VATS produced superior overall outcomes compared with chest drainage and fibrinolysis, although the included trials were small [50].

The Cochrane review by Redden and colleagues included eight randomized trials, six of which involved children. It found no significant difference in mortality or procedural complications between surgical and nonsurgical management. VATS may reduce hospital length of stay compared with thoracostomy drainage, but the review considered the evidence insufficient to define the independent contribution of fibrinolytic treatment with confidence [51].

A later pediatric systematic review and meta-analysis by Pacilli and Nataraja found similar perioperative complication rates with VATS and chest drainage with fibrinolysis. VATS was associated with a shorter post-intervention hospital stay and a lower risk of reintervention, but substantial heterogeneity and the inclusion of nonrandomized studies limited the certainty of these findings [5].

An earlier meta-analysis by Avansino and colleagues favored primary operative treatment for several short-term outcomes, including reintervention and hospitalization [52]. However, it was based predominantly on retrospective studies published before the major pediatric randomized trials and was criticized for the limited quality of its underlying evidence.

The most appropriate overall interpretation is therefore that both VATS and chest drainage with fibrinolysis are effective. Randomized trials have generally shown comparable clinical recovery, while some pooled analyses suggest that VATS may shorten post-treatment hospitalization and reduce repeat interventions. Fibrinolysis avoids general anesthesia and an initial operative procedure and is often less costly, but a minority of children will require rescue surgery. Treatment should consequently be individualized according to the child’s clinical condition, disease stage, available expertise, and response to initial drainage rather than dictated by a single universally superior strategy [5,35,45,46,47,48,49,50,51,52].

## 10. Predictors of Response to Fibrinolytic Therapy

Identifying children most likely to respond to intrapleural fibrinolysis remains an important but unresolved area of investigation. The biological rationale suggests that fibrinolysis should be most effective during the fibrinopurulent phase, when septations consist predominantly of fibrin and before extensive collagen deposition and mature pleural organization have developed [53]. However, clinical staging is imprecise, and neither symptom duration nor imaging findings can reliably determine the histological stage of disease.

A large multicenter retrospective cohort evaluated 314 children treated initially with chest-tube drainage and intrapleural fibrinolysis. Treatment failure, defined as the need for repeat pleural drainage or a total hospital stay exceeding 14 days, occurred in approximately one-third of patients. Positive blood culture and immediate admission to an intensive-care unit were independently associated with a greater risk of failure. Surprisingly, the absence rather than the presence of complex septations on baseline ultrasonography was also associated with an unfavorable outcome [53]. In this study, “complex septations” referred to multiple fibrinous strands producing a multiloculated appearance on baseline ultrasonography. Their absence did not necessarily indicate a completely anechoic or uncomplicated effusion: some children may still have had echogenic debris, isolated strands, or less extensive septation that did not meet the study’s definition of complex septations [53]. However, these lesser sonographic abnormalities were not reported separately, and the retrospective classification did not permit determination of the amount or character of debris present in the group without complex septations. The apparently greater risk of treatment failure in this group should therefore be interpreted cautiously and should not be taken to mean that sonographically simple collections are intrinsically more severe or less responsive to fibrinolysis.

Routine laboratory indices, including white blood cell count, erythrocyte sedimentation rate, C-reactive protein, albumin concentration, and urea-to-creatinine ratio, were not independently associated with treatment failure. Similarly, radiographic evidence of pulmonary necrosis on the initial chest radiograph did not reliably predict outcome [53]. These findings indicate that conventional inflammatory markers and simple radiographic characteristics should not be used in isolation to determine whether a child should receive fibrinolysis or primary surgery.

Appropriate catheter positioning and maintenance of drain patency remain clinically important because fibrinolytic agents cannot evacuate locules that are inaccessible to the chest tube [1]. Persistent sepsis may therefore reflect inadequate catheter position, catheter obstruction, an undrained collection, bronchopleural fistula, necrotizing pneumonia, or advanced pleural organization rather than pharmacological failure alone [1].

The frequently cited suggestion that children presenting within seven to ten days respond more favorably than those with longer symptom duration is biologically plausible but has not been consistently validated in pediatric studies. Likewise, pleural thickening, collection volume, and the number of septations have not demonstrated sufficient predictive accuracy to guide treatment selection reliably. Thoracic ultrasound is superior to chest radiography for defining pleural fluid complexity and guiding catheter placement, but its ability to predict fibrinolytic failure remains limited.

Consequently, no single clinical, biochemical, or imaging variable can presently identify all children who will fail fibrinolysis. Decisions should instead be based on the child’s overall severity of illness, respiratory status, microbiological findings, adequacy of drainage, and clinical response during the first 48–72 h of treatment [1,53].

## 11. Long-Term Outcomes

The long-term prognosis of pediatric pleural empyema is generally favorable. Prospective follow-up studies have shown that clinically important symptoms may persist during the first weeks or months after discharge, but most previously healthy children subsequently experience substantial or complete recovery [54].

In a prospective cohort of children assessed after pleural empyema, the majority had normal health-related quality of life, no important persistent respiratory symptoms, and satisfactory radiographic and pulmonary function outcomes at long-term follow-up. Residual radiographic abnormalities were more common during the early recovery period but resolved in most patients over time [54].

Longer-term studies using spirometry, exercise testing, radiography, and magnetic resonance imaging have similarly demonstrated good clinical recovery and predominantly normal pulmonary function. Mild residual pleural thickening, radiographic abnormalities, or limited physiological impairment may persist in a minority of children, but these findings are rarely associated with substantial functional disability [55].

More recent investigations have introduced sensitive tests such as the lung-clearance index and have detected subtle ventilation inhomogeneity in some children whose conventional spirometry was normal. These findings suggest that the term “complete recovery” should be used cautiously, because subclinical abnormalities may persist even when children are asymptomatic and standard lung-function tests are normal. Nevertheless, chronic restrictive lung disease, recurrent empyema, and severe long-term respiratory disability remain uncommon in previously healthy children [54,55].

The primary therapeutic objective should therefore be resolution of sepsis, restoration of adequate ventilation and pleural drainage, and minimization of procedural morbidity. Complete radiographic normalization during the acute admission is neither necessary nor expected, and persistent pleural opacity alone should not be interpreted as treatment failure.

## 12. International Guideline Recommendations

Evidence from randomized trials, observational cohorts, and systematic reviews has informed several national and international recommendations for the management of pediatric pleural infection. Although some differences remain regarding the preferred fibrinolytic agent and the timing of surgical referral, contemporary guidance recognizes chest-tube drainage with intrapleural fibrinolysis as an appropriate first-line strategy for many children with complicated parapneumonic effusion or empyema requiring drainage [5,51].

Management should be individualized according to the child’s respiratory status, degree of systemic illness, size and complexity of the pleural collection, presence of purulent fluid, local expertise, and response to initial treatment. Multidisciplinary collaboration involving pediatricians, respiratory physicians, infectious-disease specialists, interventional radiologists, and pediatric surgeons is desirable, particularly when the response to drainage is inadequate.

### 12.1. British Thoracic Society Guidelines

The British Thoracic Society (BYS) guideline remains one of the most influential pediatric-specific documents on pleural infection [1]. It recommends thoracic ultrasonography in all children with suspected pleural fluid and supports image-guided small-bore chest-tube placement when drainage is required.

The BTS guideline recommends intrapleural fibrinolytic therapy for complicated parapneumonic effusions and empyema because fibrinolytics improve drainage and shorten hospitalization. Urokinase is identified as the preferred agent because it was the only fibrinolytic supported by a multicenter, double-blind pediatric randomized trial at the time the guideline was developed [1].

Routine primary thoracotomy is not recommended. Surgical consultation and VATS or open decortication should be considered when a child remains clinically unwell despite appropriate antimicrobial treatment, correctly positioned drainage, and fibrinolytic therapy. Persistent radiographic abnormalities alone are not regarded as an indication for surgery.

### 12.2. PIDS/IDSA Recommendations

The original 2011 Pediatric Infectious Diseases Society and Infectious Diseases Society of America guideline recognized both chest-tube drainage with fibrinolysis and VATS as acceptable initial interventions, with treatment choice determined largely by local expertise [56].

This guidance was updated in 2026 through a series of focused PIDS/IDSA recommendations specifically addressing pediatric parapneumonic effusion and empyema. The updated guideline separately evaluates observation versus pleural drainage, chest-tube drainage with fibrinolysis versus surgical debridement, thoracostomy-tube size, and tPA alone versus combined tPA and DNase [56,57]. The 2026 recommendations continue to support pleural drainage in children with large effusions, purulent collections, or moderate effusions accompanied by respiratory distress. For children requiring invasive treatment, chest-tube drainage with fibrinolysis and surgical debridement remain accepted strategies, with the choice influenced by clinical severity, local expertise, and institutional resources [56]. The updated guideline also recommends tPA alone rather than combined tPA–DNase for pediatric fibrinolysis, reflecting randomized pediatric evidence showing no additional clinical benefit from DNase [57].

### 12.3. European Recommendations

There is no single ERS or ESPID pediatric empyema guideline equivalent in scope to the BTS document. European practice recommendations are instead derived from national guidance, expert reviews, consensus documents, and broader pediatric community-acquired pneumonia recommendations [7]. These publications generally support prompt intravenous antimicrobial therapy, thoracic ultrasonography, image-guided small-bore drainage for clinically significant collections, and intrapleural fibrinolysis for complicated or loculated effusions. Surgical consultation is recommended when adequate drainage cannot be achieved or when sepsis and respiratory compromise persist despite medical treatment [7,58].

European practice therefore also favors an individualized, stepwise approach rather than automatic primary surgery for every multiloculated collection. Differences between centers largely reflect drug availability, local interventional-radiology services, and pediatric thoracic surgical expertise.

Current recommendations from the major international scientific societies are summarized in Table 3.

## 13. Imaging-Guided Decision-Making

Thoracic ultrasonography is the preferred imaging modality for characterizing pleural collections in children. It can confirm the presence of fluid, distinguish free-flowing from septated collections, identify echogenic debris and locules, estimate collection size, and guide safe catheter insertion [1,58].

Ultrasonography provides substantially more information about pleural fluid architecture than chest radiography. Chest radiographs remain useful for identifying pneumonia, estimating the extent of hemithoracic opacification, and monitoring overall pulmonary recovery, but they cannot reliably distinguish free fluid from loculated empyema or define the optimal site for drainage [58].

The claim that ultrasound findings consistently predict operative pathology or therapeutic success should be avoided. Sonographic septations reflect pleural complexity, but ultrasound cannot reliably establish the precise histological stage of empyema. Moreover, complex septations do not necessarily predict fibrinolytic failure; in one multicenter cohort, their absence was associated with a higher risk of treatment failure [53].

Routine computed tomography is not recommended for otherwise uncomplicated pediatric empyema. In a prospective imaging study, CT provided additional information about underlying lung parenchyma but did not improve prediction of hospital stay compared with ultrasound and did not alter chest-drain management in most children [59].

CT should be reserved for selected circumstances, including diagnostic uncertainty, suspected lung abscess or necrotizing pneumonia, bronchopleural fistula, congenital pulmonary malformation, unusual pleural anatomy, or failure to improve despite apparently adequate treatment. This selective approach limits ionizing-radiation exposure while preserving CT for cases in which it is likely to influence management [1,58,59].

## 14. Practical Administration of Intrapleural Fibrinolytics

Despite the increasing acceptance of intrapleural fibrinolytic therapy, no universally standardized pediatric protocol has been established. Published regimens differ in the fibrinolytic agent selected, dose, dilution volume, dwell time, frequency of administration, chest-tube size, use of suction, and criteria for surgical escalation. Treatment should therefore follow a locally agreed multidisciplinary protocol that reflects the available evidence, institutional expertise, and characteristics of the individual patient.

Contemporary pediatric practice favors image-guided small-bore pleural catheters. The 2026 PIDS/IDSA guideline conditionally recommends small-bore tubes, defined as 12 Fr or smaller, rather than tubes of 14 Fr or larger in children requiring drainage of a parapneumonic effusion or empyema [60]. This recommendation is based on very-low-certainty evidence, but smaller catheters appear to provide adequate drainage and permit fibrinolytic administration while being less invasive and generally less painful [60]. Ultrasound-guided placement is preferred because it allows selection of an accessible fluid pocket and reduces the likelihood of inserting the catheter into consolidated lung or a poorly communicating locule.

After the fibrinolytic solution has been administered, the catheter is commonly flushed with a small volume of normal saline to deliver the full dose beyond the tubing. The drain is then clamped for a defined dwell period before free drainage or suction is resumed. Evidence supporting repeated patient repositioning is limited, although some protocols use positional changes in an attempt to improve drug distribution.

### 14.1. Urokinase Protocols

The most widely cited pediatric urokinase regimen is derived from the randomized trial conducted by Thomson and colleagues. Infants younger than one year received 10,000 IU diluted in 10 mL of normal saline, whereas children aged one year or older received 40,000 IU diluted in 40 mL [6]. The drug was administered through the pleural catheter every 12 h for three days, producing a total of six doses. The commonly used pediatric urokinase regimen should be regarded as empirically derived rather than formally dose-optimized. Although it was subsequently evaluated in a randomized clinical trial, we found no evidence that it had first undergone conventional phase 1 dose-ranging and phase 2 dose-selection studies. Accordingly, the age-based doses currently used in practice reflect historical clinical experience and trial adoption rather than a systematically established minimum effective or optimal dose.

After each administration, the drain was clamped for approximately four hours in the original trial protocol, rather than one hour as is sometimes stated in secondary summaries. The catheter was subsequently reopened to underwater-seal drainage, with suction applied according to local practice [6]. The British Thoracic Society guideline later adopted this age-based urokinase regimen while acknowledging that practical details may vary among institutions.

Clinical improvement may become apparent during the first 24–48 h, but failure should not be determined by drainage volume alone. Fever, respiratory effort, oxygen requirement, systemic inflammatory response, drain position, and the presence of residual undrained collections should all be considered.

### 14.2. Alteplase Protocols

Greater heterogeneity exists in pediatric alteplase dosing. Published regimens include fixed doses of 2 or 4 mg and weight-based doses near 0.1 mg/kg, usually subject to a maximum dose of approximately 4 mg. Higher-dose protocols, including doses up to 0.4 mg/kg or 10 mg, have also been reported, but they are not supported by evidence of superior efficacy and may be associated with more pain, oxygen desaturation, and bleeding-related concerns [40].

In the randomized trial by St Peter and colleagues, 4 mg of alteplase diluted in 40 mL of normal saline was administered at chest-tube placement and repeated at 24 and 48 h. The tube was clamped for one hour after each dose [35]. Other protocols use approximately 0.1 mg/kg diluted in 10–50 mL of saline, with a dwell time ranging from 30 min to one hour. Once-daily administration for three doses is the best studied schedule, although institutional variation remains substantial.

No pediatric trial has established that higher alteplase doses are more effective than lower-dose regimens. A conventional fixed-dose or weight-adjusted approach is therefore reasonable, provided that the dose, dilution, dwell time, and drainage procedure are standardized within the treating institution.

### 14.3. Monitoring During Therapy

Children receiving intrapleural fibrinolytic treatment require regular clinical reassessment. Monitoring should include respiratory rate and effort, oxygen requirement, temperature, hemodynamic status, pain, chest-tube patency, drainage volume and appearance, and evidence of bleeding at the insertion site or elsewhere.

Serial C-reactive protein and complete blood-count measurements may assist in assessing the overall response, but laboratory trends should not override the clinical condition. Persistent fever alone during the first days of treatment does not necessarily represent failure, particularly when respiratory status, drainage, and inflammatory markers are improving.

Direct pediatric pharmacokinetic data are limited, and systemic absorption should not be assumed to be absent. Nevertheless, clinically important systemic coagulation disturbances have been uncommon in conventional-dose pediatric series [1]. Routine coagulation testing after every dose is not generally necessary in an otherwise healthy child without bleeding risk factors. Baseline and repeat testing are appropriate in patients with thrombocytopenia, hepatic dysfunction, suspected coagulation disorders, therapeutic anticoagulation, active bleeding, or an unexplained decline in hemoglobin.

Repeat thoracic ultrasonography is appropriate when clinical improvement is inadequate, drainage suddenly ceases, catheter displacement is suspected, or a residual collection may require additional drainage. Routine daily ultrasound is not required in a child who is improving.

### 14.4. Indications for Escalation to Surgery

An incomplete radiographic response should not, by itself, be considered treatment failure. Pleural thickening, consolidation, and residual opacity may persist for weeks or months despite resolution of infection and satisfactory functional recovery.

Surgical consultation is appropriate when a child develops worsening respiratory compromise, persistent systemic toxicity, or ongoing sepsis despite appropriate antibiotics and apparently adequate pleural drainage. Escalation should also be considered when the catheter is correctly positioned but drainage remains inadequate, when an inaccessible collection persists, or when complications such as bronchopleural fistula, pulmonary necrosis, trapped lung, or recurrent catheter obstruction prevent clinical recovery [1].

A reassessment interval of approximately 48–72 h after effective drainage is commonly used, but surgery should not be delayed in a clinically deteriorating child. Conversely, persistent fever within this interval does not mandate VATS when other clinical indicators are improving.

Randomized pediatric trials have not shown clearly superior overall recovery with primary VATS compared with standardized fibrinolysis. The 2026 PIDS/IDSA guideline consequently suggests chest-tube drainage with intrapleural fibrinolysis rather than primary surgical debridement in most children who require pleural drainage, while recognizing that the certainty of evidence is very low [56]. VATS remains appropriate for refractory disease, extensive organization, complications, or situations in which effective catheter drainage cannot be achieved.

### 14.5. Complications of Intrapleural Fibrinolysis

The most frequently reported adverse effect of intrapleural fibrinolysis is transient pain or discomfort during and after instillation. Analgesia should be prescribed proactively, particularly when alteplase is used. Transient oxygen desaturation may occur because of pain, coughing, changes in intrathoracic pressure, or rapid movement of pleural fluid [33].

Low-grade fever has occasionally been reported after fibrinolytic administration, although it is difficult to distinguish a drug-related reaction from fever caused by the underlying infection. Fever and allergic reactions have historically been more frequent with streptokinase because it is a bacterial and potentially immunogenic protein [27].

Blood-stained pleural drainage is relatively common and is usually mild. Treatment need not be stopped solely because the drainage becomes lightly hemorrhagic, provided that the child remains hemodynamically stable and hemoglobin does not decline significantly.

Major hemorrhage appears uncommon but cannot be assigned a reliable universal incidence. Pediatric studies differ in dose, agent, monitoring, and the definition of clinically important bleeding. In a retrospective series using relatively large alteplase doses, 12% of children received a blood transfusion during admission, although the study could not establish that every transfusion was caused by alteplase [33]. A sudden increase in heavily bloody drainage, hemodynamic instability, bleeding at another site, or an unexplained fall in hemoglobin should prompt suspension of treatment and urgent reassessment.

### 14.6. Contraindications

Intrapleural fibrinolysis should generally be avoided in children with active major bleeding, severe uncorrected coagulopathy, clinically important thrombocytopenia, or a previous serious hypersensitivity reaction to the selected agent. Recent major surgery accompanied by ongoing or inadequately controlled bleeding is also a strong contraindication.

Therapeutic anticoagulation, congenital bleeding disorders, severe hepatic dysfunction, recent invasive procedures, extensive necrotizing pulmonary disease, and a recent major hemorrhagic event should be treated as relative contraindications requiring individualized assessment [61]. Evidence supporting rigid numerical platelet or coagulation thresholds in children is lacking. The decision should balance the likelihood of successful pleural drainage against the bleeding risk and the feasibility of alternative treatment.

### 14.7. Practical Treatment Algorithm

Management begins with prompt antimicrobial therapy directed against the likely bacterial pathogens and adjusted when microbiological results become available. Children with small, uncomplicated effusions and minimal respiratory symptoms may generally be treated with antibiotics alone. Drainage is indicated for purulent pleural fluid, large collections, moderate effusions associated with respiratory distress, or ongoing clinical instability [62].

When drainage is required, an ultrasound-guided small-bore catheter should usually be selected. Intrapleural urokinase or alteplase is appropriate when the collection is purulent, loculated, or otherwise unlikely to drain effectively through the catheter alone. The catheter should be checked regularly for obstruction and displacement. In the absence of a universally standardized pediatric protocol, urokinase may be preferred where available because it is supported by the strongest pediatric randomized evidence and by a clearly defined trial-tested regimen. Alteplase is a reasonable alternative when urokinase is unavailable, but its use should follow a standardized local protocol based on conventional fixed or weight-adjusted dosing rather than higher-dose regimens, which have not demonstrated superior efficacy and may raise additional safety concerns. These recommendations should nevertheless be interpreted cautiously because neither agent has undergone formal pediatric dose-ranging and dose-optimization studies.

Clinical response should be reassessed during the following 48–72 h. Improvement is indicated by reduced respiratory effort and oxygen requirement, resolution of systemic toxicity, declining fever burden, improving inflammatory markers, and effective drainage. When progress is unsatisfactory, repeat ultrasonography should assess drain location, residual locules, and the need for catheter replacement or an additional drain.

VATS should be considered when adequate drainage cannot be established, when sepsis or respiratory compromise persists despite appropriate treatment, or when organized pleural disease or a complication prevents lung re-expansion. This stepwise approach places effective drainage and fibrinolysis before surgery in most children while preserving prompt operative management for refractory or complicated disease [56,62].

Representative pediatric intrapleural fibrinolytic protocols reported in the literature are summarized in Table 4.

**Figure 2:**
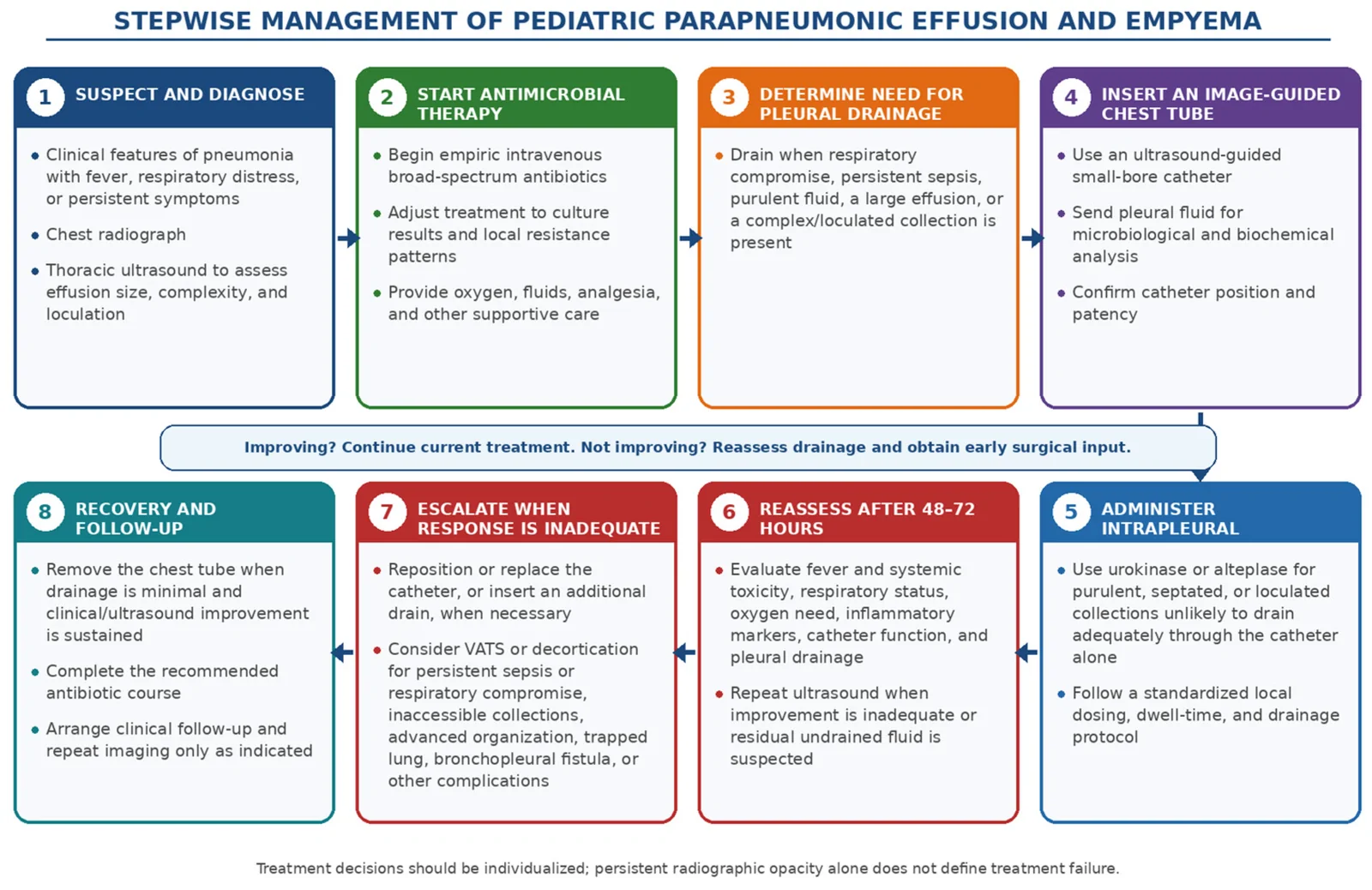
A graphical algorithm for the management of pediatric parapneumonic effusion and empyema.

The overall management of pediatric parapneumonic effusion and empyema can be summarized as a stepwise clinical algorithm integrating diagnosis, antimicrobial therapy, image-guided drainage, intrapleural fibrinolysis, reassessment, and surgical escalation when necessary (Figure 2).

## 15. Future Perspectives

Although intrapleural fibrinolytic therapy is an established component of pediatric empyema management, several important questions remain unresolved. The pediatric evidence base is derived from a relatively small number of randomized trials, together with retrospective cohorts and institutional case series. Optimal dosing has not been established through formal pediatric dose-escalation or dose-ranging studies, and currently used urokinase and alteplase regimens are largely derived from empirical practice, small trials, and heterogeneous observational series. Future research should therefore include phase 1 studies to define pharmacokinetics, systemic exposure, dose-limiting toxicity, and the minimum effective dose, followed by phase 2 trials designed to identify standardized regimens for subsequent comparative evaluation.

Patient selection also remains imprecise. No single clinical, laboratory, microbiological, or imaging variable reliably predicts which children will respond to fibrinolysis or require surgical intervention. Prospective multicenter studies should evaluate integrated prediction models incorporating disease severity, symptom duration, ultrasound characteristics, catheter position, microbiological findings, and early clinical response. Candidate biomarkers of pleural organization and impaired fibrinolysis, including plasminogen activator inhibitor-1 and selected inflammatory mediators, require specific pediatric validation before they can be used to guide treatment.

Emerging therapeutic strategies should also be assessed cautiously. Although combined tPA and DNase is effective in selected adults, current pediatric randomized evidence does not support routine DNase use. Other approaches, including alternative plasminogen activators, modified fibrinolytic agents, pleural lavage, and biomarker-guided treatment, remain investigational. Progress will require harmonized outcome definitions, standardized reporting of bleeding and treatment failure, and adequately powered multicenter trials directly comparing fibrinolytic agents, dosing strategies, and stepwise medical and surgical pathways.

Further multicenter pediatric studies are therefore needed to determine the optimal fibrinolytic agent, dose, dwell time, number of administrations, catheter-management strategy, and criteria for escalation to surgery [63]. Future studies should include formal phase 1 dose-escalation and dose-ranging trials of intrapleural fibrinolytic agents in children. Such studies would help define the minimum effective dose, dose-limiting toxicities, systemic exposure, and the relationship between dose, pleural drainage, clinical response, and bleeding risk. These data could then inform phase 2 dose-selection studies and support more standardized, evidence-based pediatric regimens rather than continued reliance on empirically derived protocols.

One area of particular interest has been the combination of tPA with recombinant human DNase. The rationale for combined treatment reflects the different components of infected pleural fluid. Fibrinolytic agents promote plasmin-mediated degradation of fibrinous septations, whereas DNase hydrolyzes extracellular DNA released predominantly from degenerating neutrophils. This extracellular DNA contributes substantially to the viscosity of purulent pleural fluid and may impede catheter drainage [38].

In adults, the MIST2 randomized trial demonstrated that combined intrapleural tPA and DNase improved radiographic pleural clearance, reduced surgical referral, and shortened hospitalization compared with placebo. Neither tPA alone nor DNase alone produced the same overall benefit [24]. These findings established combined enzyme therapy as an important option in adult pleural infection but could not be assumed to apply directly to children, whose microbiology, comorbidity burden, natural history, and probability of complete recovery differ substantially from those of adults.

Initial pediatric experience consisted of small case series and a pilot study suggesting that combined tPA–DNase administration was feasible and generally well tolerated [39]. A subsequent multicenter randomized clinical trial, however, compared three daily doses of intrapleural tPA followed by DNase with tPA followed by saline in 97 children. The addition of DNase did not reduce hospital length of stay, time to chest-tube removal, fever duration, additional pleural procedures, readmission, or healthcare costs [2]. Current pediatric evidence therefore does not support routine addition of DNase to tPA. Future studies may examine whether particular subgroups with unusually viscous, refractory, or extensively loculated collections could benefit, but such use remains investigational.

Another area of research concerns biomarkers of pleural organization and treatment response. Plasminogen activator inhibitor-1 has attracted particular attention because it suppresses endogenous fibrinolysis and promotes persistence of fibrin within the pleural space. In an adult prospective cohort, pleural fluid PAI-1 concentration was independently associated with the presence and severity of sonographic septations and with longer hospitalization. It was also associated with mortality, although it did not reliably predict the need for surgery [23]. These observations support the biological importance of PAI-1 but require prospective pediatric validation before the marker can be used to guide treatment.

Experimental work has also raised the possibility that modification of the urokinase–PAI-1 pathway could improve fibrinolytic efficacy. At present, however, PAI-1 inhibition remains a mechanistic and preclinical concept rather than an established treatment for pleural infection [21]. No biomarker currently provides sufficient accuracy to determine whether an individual child should receive fibrinolysis or primary surgery.

Inflammatory and angiogenic mediators, including interleukin-6, interleukin-8, tumor necrosis factor-α, transforming growth factor-β, and vascular endothelial growth factor, have been detected at increased concentrations in complicated parapneumonic effusions. Some adult studies have associated these mediators with pleural loculation, impaired fibrinolytic activity, or treatment failure [64]. Their clinical utility in pediatric empyema remains uncertain, and they should currently be regarded as research biomarkers rather than tools for routine risk stratification.

Advances in molecular microbiology are more immediately applicable to pediatric practice. Conventional cultures are frequently negative because antimicrobial therapy is often started before pleural fluid sampling. Molecular techniques can identify bacterial DNA in culture-negative fluid and substantially increase detection of Streptococcus pneumoniae and its serotypes. In an Australian pediatric surveillance study, pneumococcal PCR identified substantially more infections than pleural fluid culture and provided additional serotype information [65]. Molecular methods may therefore improve antimicrobial targeting and surveillance of changing pneumococcal epidemiology.

Broad-range 16S ribosomal RNA sequencing and other next-generation molecular techniques may further increase diagnostic yield, particularly in culture-negative or polymicrobial infections. Their routine clinical role remains limited by cost, availability, turnaround time, contamination risk, and uncertainty regarding the significance of detecting nonviable bacterial DNA.

Prediction models integrating clinical severity, microbiology, laboratory findings, and imaging could eventually improve patient selection. At present, however, no artificial-intelligence or machine-learning model has been prospectively validated for predicting fibrinolytic failure in pediatric empyema. Discussion of such methods should therefore be framed as a possible future research direction rather than an emerging clinical application.

Substantial uncertainty also remains regarding the optimal fibrinolytic regimen. Pediatric practice varies in the choice between urokinase and alteplase, fixed versus weight-based dosing, dilution volume, dwell time, frequency of administration, and total treatment duration. Existing alteplase protocols are based predominantly on observational experience, while the most widely cited urokinase regimen derives from a single multicenter randomized trial [40,63]. Direct pediatric comparisons of urokinase and alteplase are lacking.

The ideal timing of intervention also remains unclear. Fibrinolysis is biologically most likely to be effective before extensive collagen deposition and mature pleural organization occur. A comparative pediatric study reported greater success in fibrinopurulent than chronic-stage empyema, supporting the concept that advanced organization reduces responsiveness to pharmacological treatment [66]. Nevertheless, symptom duration and imaging appearances do not accurately define the histological stage, and no prospective trial has established a precise therapeutic window.

Fibrinolysis and surgery should be considered complementary rather than mutually exclusive treatments. Chest-tube drainage with fibrinolysis provides a minimally invasive first-line option for many children, while VATS remains valuable when drainage cannot be established, clinical sepsis persists, or advanced pleural organization prevents lung re-expansion. Randomized trials have generally shown similar clinical recovery with both strategies, although a minority of children treated initially with fibrinolysis require rescue surgery [5].

Economic evaluations have generally favored fibrinolysis. In the randomized trial by St Peter and colleagues, primary VATS did not improve clinical recovery but resulted in substantially higher hospital charges than chest-tube drainage with alteplase [35]. Sonnappa and colleagues similarly found urokinase-based treatment to be less costly than primary VATS, with no significant difference in the principal clinical outcomes [45]. Cost comparisons nevertheless depend on local operating-room expenses, interventional-radiology resources, hospital organization, and thresholds for repeat intervention.

Because individual centers encounter relatively small numbers of severe cases, future progress will depend on multicenter collaboration. Prospective registries and harmonized clinical datasets could facilitate adequately powered comparisons of fibrinolytic agents, dosing strategies, imaging classifications, microbiological methods, patient-reported outcomes, and long-term pulmonary function. Standardized definitions of treatment success, fibrinolytic failure, bleeding, and surgical escalation will be essential to reduce heterogeneity and improve comparison across studies [53].

## 16. Conclusions

Pediatric pleural empyema remains an important cause of morbidity among children hospitalized with community-acquired pneumonia. Timely antimicrobial therapy, accurate imaging, and effective pleural drainage are essential to control infection, relieve respiratory compromise, and reduce the need for more invasive intervention.

Current evidence supports intrapleural fibrinolytic therapy as an effective first-line adjunct to chest-tube drainage in many children with complicated parapneumonic effusion or empyema. Fibrinolytic drugs promote degradation of accessible fibrinous septations, improve communication among pleural locules, and facilitate drainage through an appropriately positioned catheter.

Urokinase is supported by a multicenter placebo-controlled pediatric trial, whereas alteplase is supported by observational cohorts and randomized comparisons with VATS. Both agents are widely used, but no direct randomized pediatric comparison has established that one is superior. Serious bleeding appears uncommon with conventional-dose regimens, although the precise incidence cannot be defined because doses and adverse-event reporting vary across studies.

Randomized trials comparing fibrinolysis with VATS have generally demonstrated similar hospital recovery, fever duration, oxygen requirements, and long-term outcomes. Fibrinolysis avoids an initial operation and is often less costly, whereas VATS may provide more immediate mechanical clearance and may reduce repeat intervention in some populations. Treatment selection should therefore reflect the child’s clinical condition, the adequacy of catheter drainage, local expertise, and the response to initial therapy.

Management is best delivered through a multidisciplinary, stepwise pathway incorporating prompt antibiotics, thoracic ultrasonography, image-guided small-bore catheter drainage, appropriate intrapleural fibrinolysis, and timely surgical consultation. Failure of fibrinolysis does not necessarily represent inappropriate initial management, provided that inadequate response is recognized promptly and rescue intervention is not unduly delayed.

The most important remaining research priorities include direct comparison of urokinase and alteplase, optimization of pediatric dosing, validation of biomarkers of pleural organization, improved microbiological diagnosis, and standardization of criteria for treatment failure. Current evidence does not support routine addition of DNase to tPA in children.

Overall, intrapleural fibrinolysis is a safe and effective treatment strategy for appropriately selected children with pleural empyema. When used as part of an organized treatment pathway, it permits most patients to recover without primary surgery while preserving VATS as an effective option for refractory or advanced disease.

## Figures and Tables

**Table 1:** Comparison of Available Intrapleural Agents.

Agent	Mechanism	Advantages	Limitations	Pediatric Evidence
Streptokinase	Indirect plasminogen activation	Low historical cost	Antigenic, non-fibrin specific	Limited; not routinely recommended
Urokinase	Direct plasminogen activator	Low immunogenicity, most pediatric data	Availability varies	Highest-quality pediatric RCT
Alteplase (tPA)	Recombinant tPA	Widely available	Variable dosing	Strong observational evidence + RCT vs. VATS
Tenecteplase	Modified tPA	Longer half-life	Very limited evidence	Investigational
DNase *	Extracellular DNA degradation	Reduces pus viscosity	Not a fibrinolytic	No routine pediatric use

Abbreviations: * DNase is an adjunctive enzymatic agent rather than a fibrinolytic. RCT, randomized controlled trial; tPA, tissue plasminogen activator; VATS, video-assisted thoracoscopic surgery.

**Table 2:** Landmark Clinical Studies.

Study	Design	Intervention	Comparator	Main Findings
Thomson 2002	Multicenter RCT	Urokinase	Saline	Shorter hospital stay
Sonnappa 2006	RCT	Urokinase	VATS	Comparable outcomes; lower cost
St Peter 2009	RCT	Alteplase	VATS	Comparable recovery; lower cost
Marhuenda 2014	Multicenter RCT	Urokinase	VATS	No significant differences
Livingston 2020	Multicenter RCT	tPA + DNase	tPA	No added benefit of DNase

Abbreviations: RCT, randomized controlled trial; VATS, video-assisted thoracoscopic surgery; tPA, tissue plasminogen activator; DNase, deoxyribonuclease.

**Table 3:** International Guideline Recommendations.

Guideline	Drainage	Fibrinolysis	Surgery	Preferred Agent
BTS 2005	Yes	Yes	Rescue	Urokinase
PIDS/IDSA 2011	Yes	Acceptable	Acceptable	None
PIDS/IDSA 2026	Small-bore tube	Preferred before surgery	Rescue	tPA
European	Yes	Loculated effusions	Individualized	Institution-dependent

Abbreviations: BTS, British Thoracic Society; PIDS, Pediatric Infectious Diseases Society; IDSA, Infectious Diseases Society of America; tPA, tissue plasminogen activator.

**Table 4:** Practical Pediatric Fibrinolysis Protocols.

Parameter	Urokinase	Alteplase
Dose	<1 yr:10,000 IU; ≥1 yr:40,000 IU	0.1 mg/kg (max 4 mg) or fixed 2–4 mg
Dilution	10–40 mL saline	20–50 mL saline
Dwell time	~4 h	30–60 min
Frequency	Every 12 h	Usually once daily
Duration	3 days (6 doses)	3 doses
Evidence	Pediatric RCT	Observational + RCT vs. VATS

Abbreviations: yr, year; IU, international units; mg, milligram; kg, kilogram; mL, milliliter; h, hour; min, minutes; max, maximum; RCT, randomized controlled trial; VATS, video-assisted thoracoscopic surgery.

## Data Availability

No new data was created or analyzed in this study. Data sharing is not applicable to this article.
